# Susceptibility of* In Vitro* Melanoma Skin Cancer to Photoactivated Hypericin versus Aluminium(III) Phthalocyanine Chloride Tetrasulphonate

**DOI:** 10.1155/2017/5407012

**Published:** 2017-09-25

**Authors:** I. M. Ndhundhuma, H. Abrahamse

**Affiliations:** Laser Research Centre, Faculty of Health Sciences, University of Johannesburg, Doornfontein 2028, South Africa

## Abstract

The sensitivity of human melanoma cells to photoactivated Hypericin (Hyp) compared to aluminium(III) phthalocyanine chloride tetrasulphonate (AlPcS_4_Cl) is reported in this study. Melanoma cells (A375 cell line) were treated with various concentrations of Hyp or AlPcS_4_Cl alone, for 1, 4, and 24 hrs; varying doses of laser irradiation alone (594 or 682 nm); or optimal concentrations of PSs combined with laser irradiation. Changes in cell morphology, viability, membrane integrity, and proliferation after treatment of cells were determined using inverted microscopy, Trypan blue cell exclusion, Lactate Dehydrogenase (LDH) membrane integrity, and adenosine triphosphate (ATP) cell proliferation assay, respectively. More than 60% of cell survival was observed when cells were treated with 2.5 *μ*M of Hyp or AlPcS_4_Cl alone at all incubation times or with 5 J/cm^2^ of 594 or 682 nm laser alone. Combination of PSs and respective lasers leads to a statistically significant incubation time-dependent decrease in survival of cells. Flow cytometry using the FITC Annexin V/PI apoptosis kit demonstrated that cell death induced after Hyp-PDT is via early and late apoptosis whereas early apoptosis was the main mechanism observed with AlPcS_4_Cl-PDT. Hyp-PDT compared to AlPcS_4_Cl-PDT is indicated to be a more effective cancer cell death inducer in melanoma cells.

## 1. Introduction

Excessive exposure of skin to ultraviolet radiation can damage cellular DNA leading to skin cancer [1, 2]. Other factors that cause skin cancer include skin exposure to carcinogens or having a condition that weakens the immune system [3]. Nonmelanoma skin cancers are curable if detected early [4]. However, melanoma is a rare form of skin cancer that is mostly unresponsive to conventional treatments [5]. Conventional treatments for melanoma are determined by the stage, site of melanoma, and overall health of patient [5]. Surgery is commonly used for early stage melanoma, whereas late stage melanoma may be treated with radiation, immunotherapy, targeted therapy, or chemotherapy [5]. However, these treatments often have a poor prognosis, due to metastatic melanoma resistance; thus the search for suitable cure remains ongoing [5, 6].

Photodynamic therapy (PDT) is an unconventional treatment method that has been applied to various forms of cancer and currently is an established form of treatment for nonmelanoma skin cancer. It involves the selective treatment of cancer tissues, using photosensitizer (PS) drug which has been excited with light in the presence of molecular oxygen to produce singlet oxygen and other reactive species to destroy cancer cells. The selective and easy application of photosensitizers (PSs) and light delivery to skin has led PDT to be an increasingly exploited therapy in dermatology [7]. The benefits of PDT are its selective treatment of diseased area, while preserving neighboring normal tissue and the excellent cosmetic effects after treatment [8]. 5-Aminolevulinic acid (ALA (Levulan®)); methyl 5-aminolevulinate (MAL (Metvix®)); and metatetrahydroxyphenyl chlorine (Foscan/Temoporfin) are currently approved PSs for the treatment of actinic keratosis (AK), basal cell carcinoma (BCC), and neck and head cancers [8, 9]. Side-effects of burning and stinging have been reported during treatments, and sometimes after treatment transient localized erythema, edema, and crusting have been noted [10]. However, the search for a suitable PS and PDT treatment protocol for melanoma and squamous cell carcinoma (SCC) continues [11–14]. The most significantly investigated PSs for PDT in dermatology are Hyp and AlPcS_4_Cl. Both PSs have shown great potential in melanoma treatment yet have not been approved for clinical application. Moreover,* in vitro* studies for the direct comparison of these PSs on melanoma cells in order to establish the suitable PS dose responses for melanoma treatment have not yet been reported.

An ideal PS is characterized by no dark toxicity, low tendency to form aggregates, photostability, absorption of light at longer wavelengths, production of significant amount of singlet oxygen, fluorescent, low absorbance to day light, no retention in healthy tissue, and high uptake in diseased tissue. Phthalocyanines (Pc) are synthetic dyes that have a high molar absorption coefficient in the red part of the spectrum [15]. One of the previously tested PSs, hydrophilic AlPcS_4_Cl, has been shown to be a promising PS agent in the PDT treatment of melanoma skin cells [16, 17]. On the other hand, Hyp is a lipophilic dianthraquinone with a wide absorbance spectrum [18]. It has been used for many years as an antidepressant drug and has also been reported as one of the most potent naturally occurring PDT agents [19]. The scope of this work was to directly compare the susceptibility of human malignant melanoma A375 cells to Hyp and AlPcS_4_Cl in terms of cellular toxicity, subcellular localization, and photodynamic efficacy to possibly assist in the choice and dose of the ideal photoactive PS for melanoma treatment.

## 2. Methods

### 2.1. Photosensitizers

Hydrophilic aluminium(III) phthalocyanine chloride tetrasulphonate (AlPcS_4_Cl), molecular weight 895.19 g/mol, (Frontier Scientific, Logan, UT, USA), and Hypericin, molecular weight 504.44 g/mol (Sigma-Aldrich, 56690-1 MG), were used. Stock solutions of 100 *μ*M AlPcS_4_Cl were solubilized in Dulbecco's phosphate buffered saline (DPBS; Sigma-Aldrich, D8537) and 2 mM Hypericin were solubilized in dimethyl sulfoxide (DMSO, BDM Merck, Germany) and sterilized using a 0.2 *μ*m filter.

### 2.2. Cell Culture

Cultures of human malignant melanoma (A375 cell line, ATCC number CRL-1619) were grown in the supplemented culture medium consisting of Dulbecco's modified Eagle's medium with L-glutamine and phenol red (DMEM; Sigma-Aldrich, D5796), supplemented with 10% heat-inactivated fetal bovine serum (FBS; Biochrom GmbH, S0615), 1% penicillin-streptomycin mixture (Sigma-Aldrich, P4333), and 1% Amphotericin B (Sigma-Aldrich, A2942). Cell cultures were incubated at 37°C in 5% CO_2_. When confluent, cells were washed three times with DPBS, detached using TrypLE Express™ (Gibco Invitrogen, INV 12605028, 4 to 5 ml/175 cm^2^ tissue culture flask), and seeded at 4 × 10^4^ cells/ml in 3.3 cm^2^ diameter tissue culture dishes which contained 3 ml of supplemented DMEM and were so further incubated for 24 hrs to allow for attachment.

### 2.3. PS and Laser Irradiation Dose Response

Dose-response curves using various concentrations of the PSs and different incubation times after irradiation and prior to performing biochemical assays (1, 4, and 24 hrs) were performed to determine cytotoxicity levels induced by PSs. In order to evaluate biochemical effects, a concentration of 2.5 *μ*M of both PSs, Hyp and AlPcS4Cl, which induce approximately 50% cytotoxicity (ICD50), was selected to observe cellular changes. Attached cells in culture dishes were washed twice with 1 ml DPBS and 1, 2.5, 5, and 10 *μ*M Hyp or AlPcS_4_Cl were added to 3 ml of freshly replaced supplemented DMEM. Control cells contained cell culture medium without PS. Cells with or without PS were divided into three groups. Group 1 were cells incubated in the dark for 1 hr, Group 2 were cells incubated in the dark for 4 hrs, and Group 3 were cells incubated in the dark for 24 hrs. After respective incubation periods cells were washed twice with 1 ml DPBS and 3 ml of fresh supplemented DMEM was added. All samples were then incubated for 48 hrs after treatment and changes in cell morphology, viability, proliferation, and cytotoxicity were determined, to determine the optimal concentration of each PS to be used in PDT experiments.

Two diode lasers, at wavelengths (*λ*) of 594 and 682 nm (Oriel Corporation, USA, LREBT00 ROITHI) provided by the National Laser Centre of South Africa, were used to irradiate cells. Both photosensitizer and laser treatment parameters are listed in Table 1. The laser at wavelength 594 nm was used to irradiate cells treated with Hyp, while the diode laser at wavelength of 682 nm was used to irradiate cells treated with AlPcS_4_Cl. The laser spot size covered the entire area of the culture dish (3.3 cm^2^). Prior to performing laser irradiation experiments, a FieldMate Laser Power Meter was used to measure the lasers output power in order to determine laser irradiation exposure times. All irradiation protocols were performed with the lights off so as to eliminate any other external light interference.

In laser irradiation dose-response assays cells in culture dishes were washed twice with 1 ml DPBS and then divided into two groups: Group 1, cells irradiated at a wavelength of 594 nm; Group 2, cells irradiated at a wavelength of 682 nm, with a fluence range of 1, 2, 5, or 10 J/cm^2^. Control cells in all experiments were cells without PS or not irradiated. After laser irradiation, the DPBS was removed from all the culture dishes and 3 ml of fresh supplemented DMEM was added, following an additional 48 hrs of incubation, after which, changes in cell morphology, viability, proliferation, and membrane integrity were determined, to decide upon the optimal laser dose fluence to be applied at each respective wavelength that was to be applied in PDT experiments.

### 2.4. Photodynamic Effect

Cells in culture dishes were washed twice with 1 ml DPBS and 2.5 *μ*M of either PS in 3 ml of fresh supplemented DMEM was added, as this was determined in previous experiments to be the optimal concentration of each PS to be used in these PDT experiments (Section 2.3). All culture dishes were incubated for an additional 1, 4, or 24 hrs. The culture media were then replaced with 2 ml DPBS and cells were then subjected to laser irradiation at 594 nm (for cells that contained Hyp) or 682 nm (for cells that contained AlPcS_4_Cl), at a laser dose of 5 J/cm^2^, which was found to be the optimum laser dose fluence in experiments performed above. After laser irradiation, the DPBS was removed from all the culture dishes and 3 ml of fresh supplemented DMEM was added. The culture dishes were then incubated for an additional 48 hrs. Three control groups were prepared. Group 1 was cells alone; Group 2 was cells with 2.5 *μ*M PS and not irradiated; and Group 3 included 2.5 *μ*M PS and irradiation at 5 J/cm^2^. All samples were incubated for 48 hrs after treatment and changes in cell morphology, viability, proliferation, and membrane integrity were determined.

### 2.5. Changes in Cell Morphology

Inverted light microscopy (Wirsam, Olympus CKX41) was used to observe and study cellular morphological changes. Images were digitally captured using a SC30 Olympus camera.

### 2.6. Cell Viability

Trypan blue (Sigma-Aldrich, T8154) dye exclusion viability assay was used to determine the percentage viability of untreated and treated A375 cells. 10 *μ*l Trypan blue reagent was added to 10 *μ*l of cell suspension and then loaded onto a cell counting chamber slide (Invitrogen, C10283) designed for use with Countess™ Automated Cell Counter (Invitrogen, C10227). In the Trypan blue assay, cells with an intact cellular membrane do not take up the dye and maintain a clear appearance under the microscope, while damaged cells take up the dye and so appear blue in colour.

### 2.7. Cytotoxicity

Lactate Dehydrogenase Assay Kit (Cytotox96® assay, Promega G400) was used according to the manufacturer's instructions to measure the Lactate Dehydrogenase (LDH) released from the cytosol into the cell culture media upon cell membrane damage. The membrane integrity of untreated and treated cells was assessed by estimating the amount of LDH present in the culture media. Fifty microliters of reconstituted LDH reagent was added to an equal volume of cell culture medium from untreated and treated cells and incubated in the dark at room temperature for 30 min. The calorimetric compound was measured spectrophotometrically at 490 nm using the PerkinElmer, VICTOR3™, microplate reader.

### 2.8. Cell Proliferation

The CellTiter-Glo® Luminescent Assay (Promega, G7571, Anatech Analytical Technology, Bellville, South Africa) was used according to the manufacturer's instructions to evaluate metabolically active cells by measuring ATP signal of proliferative cells. Fifty microliters of reconstituted reagent was added to an equal volume of cell suspension in a 96-well plate and mixed by placing the plate on a plate shaker for 2 minutes to induce cell lysis. The mixture was then incubated in the dark at room temperature for 10 minutes to stabilize the luminescent signal. The ATP content in each sample was quantified by recording luminescence using the PerkinElmer, VICTOR3 Multilabel Counter (model 1420) in relative light units (RLUs).

### 2.9. Mode of Cell Death

The FITC Annexin V Apoptosis Detection Kit (BD Pharmingen™, 556547) was used to detect either early/late apoptotic or necrotic modes of cell death by flow cytometry. In apoptotic cells, the membrane phospholipid phosphatidylserine, which is normally found in the internal portion of the cell membrane, becomes translocated to the outer leaflet of the plasma membrane, thereby exposing phosphatidylserine to the external environment. Annexin V is a calcium-dependent phospholipid binding protein that has an affinity for phosphatidylserine and is useful in identifying apoptotic cells, whereas Propidium Iodide (PI) is used to identify necrotic cells. A375 cells after PDT treatment were washed twice in 200 *μ*l PBS and detached using TrypLE Express (Gibco Invitrogen, INV 12605028). Cells were then resuspended in 200 *μ*l Binding Buffer (1x) at a cell density of 1 × 10^6^/ml, after which 5 *μ*l FITC Annexin V and 5 *μ*l PI were added to cell suspension and vortexed. Stained cell samples were incubated for 10 min at room temperature and 200 *μ*l of Binding Buffer (1x) was again added to cell suspension prior to analysis with flow cytometer. All experimental parameters were monitored using appropriate controls.

### 2.10. Statistical Analysis

Each set of experiments was repeated six times (*n* = 6) using melanoma cell line at passages between 15 and 20, while each biological assay was performed in triplicate. Untreated cells were compared to treated cells using Sigma Plot version 12.0 and the mean, standard deviation, and standard error were determined. Statistical significance between untreated control cells and treated cells is shown in the graphs as ^*∗*^*P* < 0.05, ^*∗∗*^*P* < 0.01, and ^*∗∗∗*^*P* < 0.001. Significant differences were considered at the 95th percentile.

## 3. Results 

### 3.1. Changes in Cell Morphology

**Figure 1:**
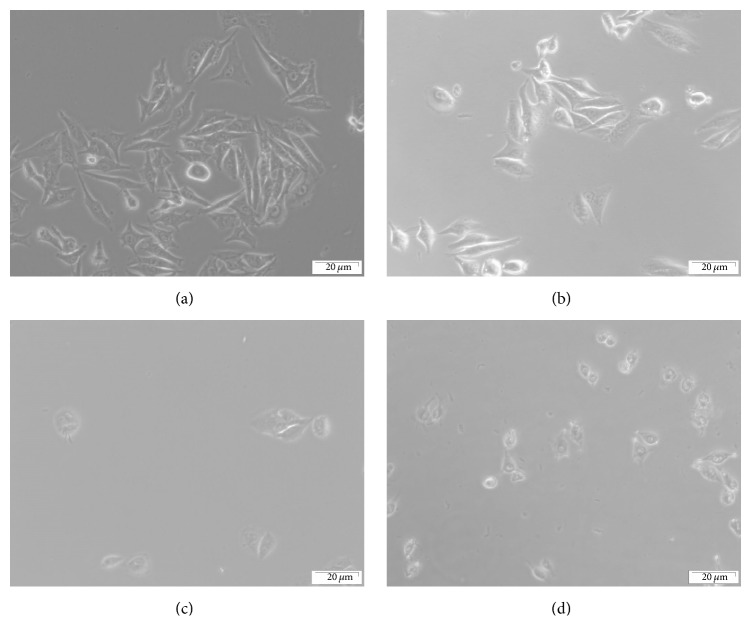
Morphological features of A375 treatment with laser irradiation at 5 J/cm^2^ or combination of laser and PS (2.5 *μ*M). (a) Laser irradiation alone at 682 nm; (b) laser irradiation alone at 594 nm; (c) combination of laser irradiation at 682 nm and 2.5 *μ*M AlPcS_4_Cl; (d) combination of laser irradiation at 594 nm and 2.5 *μ*M Hyp. Combination of laser and PS (AlPcS_4_Cl or Hyp) leads to destruction of cells.

Photochemical effects of Hyp-PDT and AlPcS_4_Cl-PDT for treatment of A375 cells* in vitro* lead to distinctive cell morphological changes and cell death. Cells irradiated with laser dose of 5 J/cm^2^ at wavelengths 594 and 682 nm showed no signs of morphological damage.  Figure 1 illustrates morphological features of A375 cells after treatment with laser irradiation at 5 J/cm^2^ or combination of cells treated with PS (2.5 *μ*M) and incubated for 24 hrs, followed by laser irradiation at 594 nm or 682 nm. Laser irradiation alone at 682 nm or 594 nm did not note any significant morphological changes. However, significant changes in cell morphology were observed when cells previously incubated with 2.5 *μ*M AlPcS_4_Cl were irradiated with laser light at 682 nm (Figure 1(c)) or with 2.5 *μ*M Hyp irradiated at 594 nm (Figure 1(d)). Figures 1(c) and 1(d) indicate that the combination of PS and laser light causes destruction of melanoma* in vitro*. Signs of cell damage, marked by shrinkage and detachment of A375 cells from the surface of the tissue culture dish, became apparent when A375 cells were incubated with PS for 24 hrs followed by irradiation with laser light.

### 3.2. Cellular Viability

The cell viability of the A375 cells was assessed by using the Trypan blue dye exclusion viability assay (TB). Trypan blue dye is a negatively charged chromophore that does not interact with intact cell membranes but with damaged cell membranes. Hence, live cells have poor affinity to Trypan blue dye, whereas dead cells have high affinity to Trypan blue. This feature is therefore used to discriminate dead amongst live cells. Untreated cells (cells without PSs and nonirradiated serve as control cells) and treated cells were compared using Trypan blue viability assay 48 hrs after treatment. Percentage cell viability of cells treated with 1, 2.5, and 5 *μ*M Hyp alone was found to be insignificantly different to untreated cells. However, those treated with 10 *μ*M Hyp showed significant differences at all incubation times (1, 4, and 24 hrs). No significant differences were observed between untreated and treated cells with 1, 2.5, 5, and 10 *μ*M AlPcS_4_Cl at all incubation times. The results to show the effect of PSs alone were not presented in this paper but can be made available upon request. In order to evaluate biochemical effects, a concentration of 2.5 *μ*M of both PSs, Hyp and AlPcS4Cl, which induce approximately 50% cytotoxicity (ICD50), was selected.

**Figure 2:**
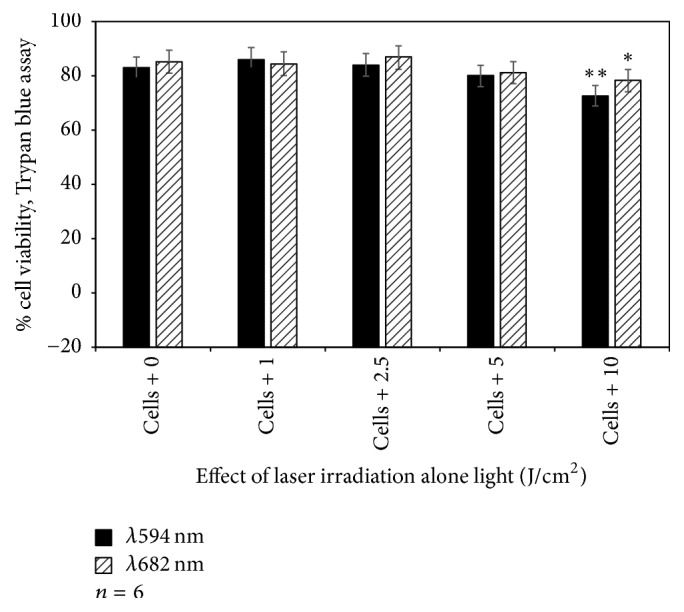
Trypan blue viability assay to evaluate effect of laser irradiation at wavelengths 594 and 682 nm on A375 cells. Untreated A375 (control: 0 J/cm^2^ of laser) were compared with those treated with 1, 2.5, 5, and 10 J/cm^2^.

Laser irradiation of A375 cell alone at 1, 2.5, and 5 J/cm^2^ showed no toxicity to A375 cells at both wavelengths (Figure 2). But statistical significant differences between untreated cells and those treated with 10 J/cm^2^ at both wavelengths were observed.

**Figure 3:**
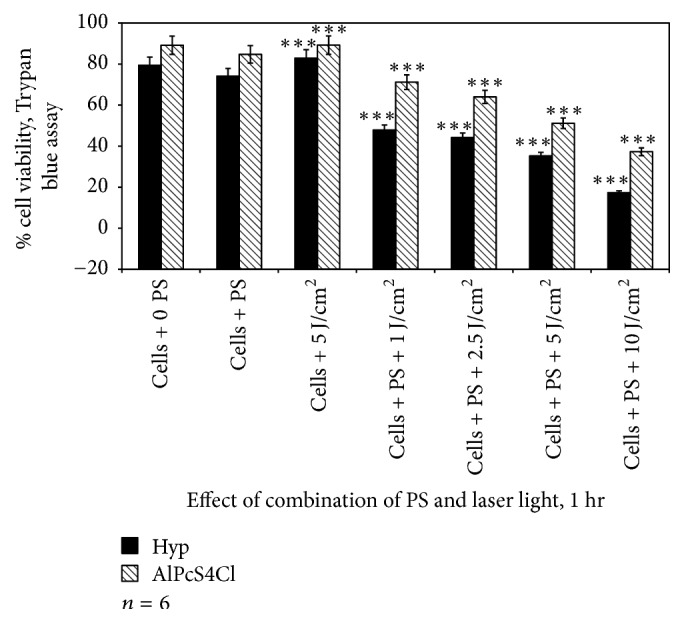
Trypan blue viability assay to assess the effect of Hyp-PDT versus AlPcS_4_Cl-PDT when cells were treated with Hyp or AlPcS_4_Cl for 1 hr followed by laser irradiation at wavelengths 594 and 682 nm, respectively. Untreated A375 (cell + 0 PS, 0 LI) were compared with those treated with 2.5 *μ*M PS, 5 J/cm^2^ and those treated with 2.5 *μ*M PS + 1, 2.5, 5, and 10 J/cm^2^.

**Figure 4:**
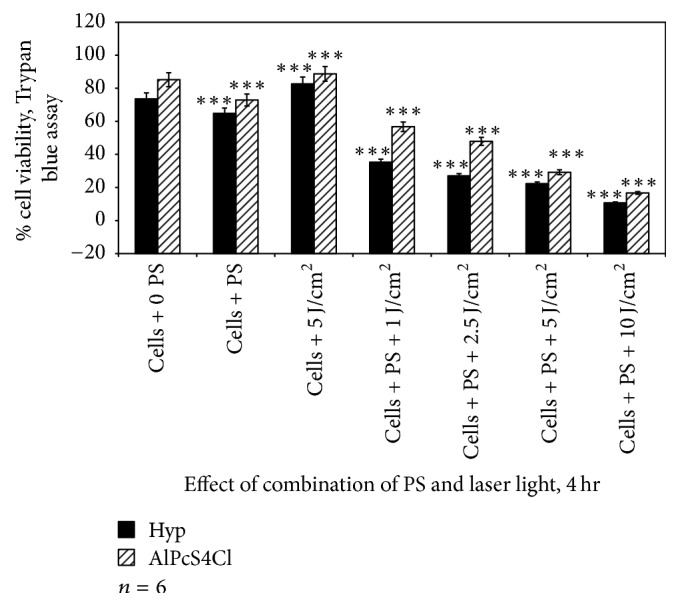
Trypan blue viability assay to assess the effect of Hyp-PDT versus AlPcS_4_Cl-PDT when cells were treated with Hyp or AlPcS_4_Cl for 4 hrs followed by laser irradiation at wavelengths 594 and 682 nm, respectively. Untreated A375 (cell + 0 PS, 0 LI) were compared with those treated with 2.5 *μ*M PS, 5 J/cm^2^, and those treated with 2.5 *μ*M PS + 1, 2.5, 5, and 10 J/cm^2^.

**Figure 5:**
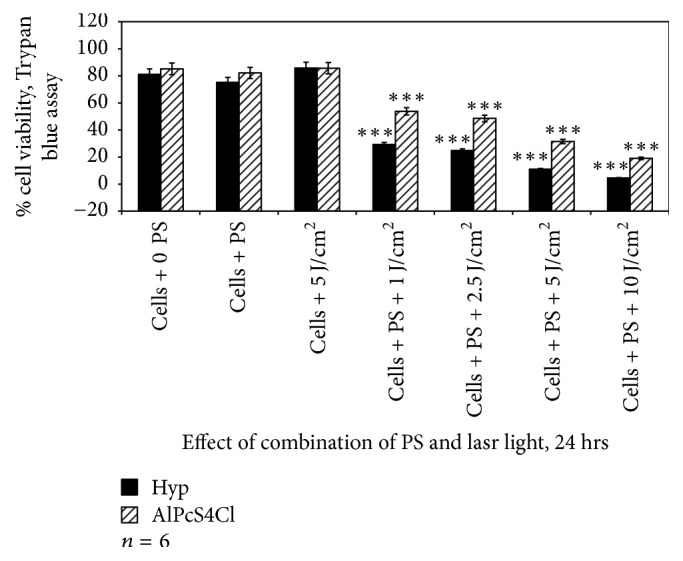
Trypan blue viability assay to assess the effect of Hyp-PDT versus AlPcS_4_Cl-PDT when cells were treated with Hyp or AlPcS_4_Cl for 24 hrs followed by laser irradiation at wavelengths 594 and 682 nm, respectively. Untreated A375 (cell + 0 PS, 0 LI) were compared with those treated with 2.5 *μ*M PS, 5 J/cm^2^ and those treated with 2.5 *μ*M PS + 1, 2.5, 5, and 10 J/cm^2^.

Irradiating A375 cells that contained 2.5 *μ*M of either Hyp or AlPcS_4_Cl at a wavelength of 594 nm or 682 nm with a laser dose of 1, 2.5, 5, or 10 J/cm^2^, respectively, resulted in a loss of cell viability (Figures 3–5). Comparing the percentage loss of cell viability when A375 were treated with Hyp-PDT versus AlPcS_4_Cl-PDT at 1 hr, the percentage loss was significant at 1, 2.5, 5, and 10 J/cm^2^ when compared to their respective control cell population receiving no irradiation or photosensitizer.

Figure 4 demonstrates the effect of PDT after treating cells with either PS for 4 hrs followed by irradiation at their selective wavelength of absorption. Comparing the percentage loss of cell viability when A375 were treated with Hyp-PDT versus AlPcS4Cl-PDT at 4 hrs, the percentage loss in viability was significantly higher for all fluences when comparing the two PSs, as well as considering the increased incubation periods.

Finally, as expected, cellular viability was severely affected in cells that were incubated with PSs for 24 hrs (Figure 5) as compared to 1 hr and 4 hrs incubation times, suggesting that the longer the incubation period of cells with both PSs, the higher the PDT effect.

### 3.3. Cytotoxicity

Incubation of A375 cells with Hyp at 1, 2.5, and 5 *μ*M exhibited no significance difference on the LDH signal between control cells and treated cells. However, at 10 *μ*M, alone statistical differences within both control and treated cells were observed, suggesting that 10 *μ*M Hyp alone is toxic to cells. No statistical significant differences were observed in the LDH signal between untreated and treated cells with 1, 2.5, 5, and 10 *μ*M AlPcS_4_Cl at all incubation times. Laser irradiation of A375 cell alone at 1, 2.5, and 5 J/cm^2^ showed no toxicity to A375 cells at either wavelengths (Table 2). However, statistical significant differences between untreated cells and those treated with 10 J/cm^2^ at 682 nm laser (*P* < 0.05) were noted. No significant differences between untreated cells and those treated with 10 J/cm^2^ at 594 nm laser were noted.

Susceptibility of cells to Hyp-PDT and AlPcS_4_Cl-PDT treatment was evaluated over a 1, 4, and 24 hrs period. LDH signal is inversely proportional to viable cell number with intact membrane integrity in culture. Loss of membrane integrity in cells was confirmed when difference in LDH signal of untreated and treated groups was statistically significant. Significant cellular damage was noted in treated cells compared to untreated cells (Table 3).

### 3.4. Cell Proliferation

The CellTiter-Glo Luminescent Cell Proliferation Assay is a robust, homogeneous, fast, and sensitive assay based on quantification of the content of ATP in cells to signal the number of metabolically energetic cells. It involves mixing a single reagent with cells in culture media to lyse cells and generating the luminescent signal that is a measure of the ATP content present in cells. A375 ATP content was evaluated to determine the level of metabolic active versus metabolically damaged cells after PDT treatment. ATP is a marker for both viability and proliferation of cells. ATP signal is directly proportional to the number of metabolically active cells.

**Figure 6:**
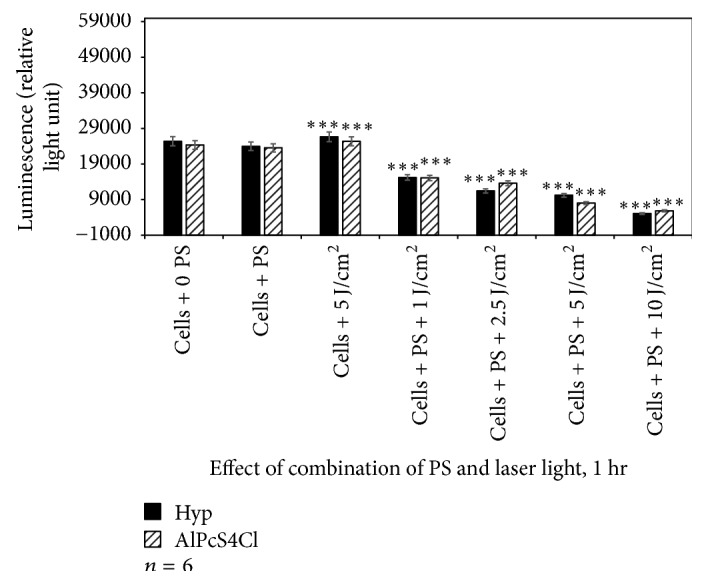
Adenosine triphosphate assay to assess the effect of Hyp-PDT versus AlPcS_4_Cl-PDT when cells were treated with Hyp or AlPcS_4_Cl for 1 hr followed by laser irradiation at wavelengths 594 and 682 nm, respectively. Untreated A375 (cell + 0 PS, 0 LI) were compared with those treated with 2.5 *μ*M PS, 5 J/cm^2^ and those treated with 2.5 *μ*M PS + 1, 2.5, 5, and 10 J/cm^2^.

**Figure 7:**
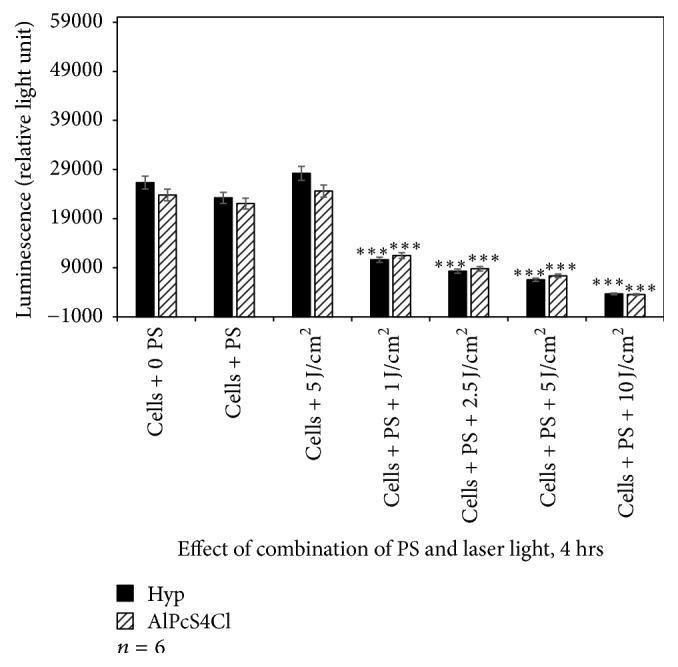
Adenosine triphosphate assay to assess the effect of Hyp-PDT versus AlPcS_4_Cl-PDT when cells treated with Hyp or AlPcS_4_Cl for 4 hrs followed by laser irradiation at wavelengths 594 and 682 nm, respectively. Untreated A375 (cell + 0 PS, 0 LI) were compared with those treated with 2.5 *μ*M PS, 5 J/cm^2^ and those treated with 2.5 *μ*M PS + 1, 2.5, 5, and 10 J/cm^2^.

**Figure 8:**
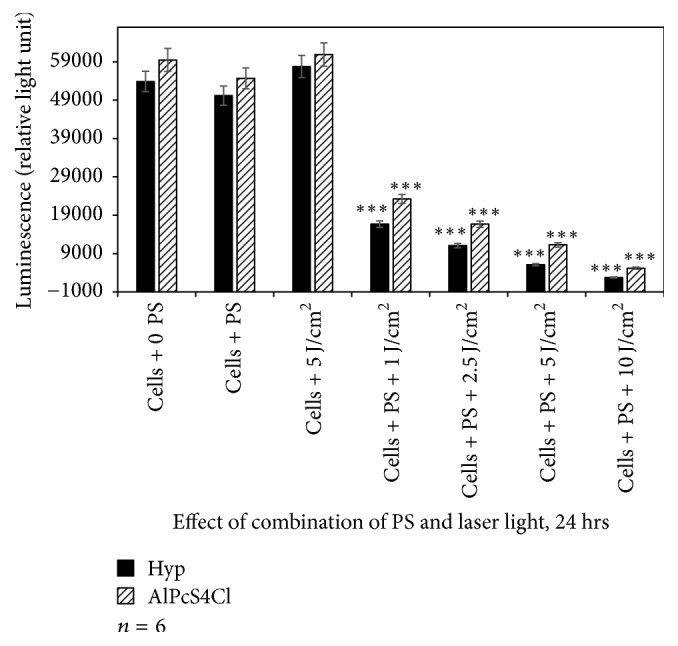
Adenosine triphosphate assay to assess the effect of Hyp-PDT versus AlPcS_4_Cl-PDT when cells were treated with Hyp or AlPcS_4_Cl for 24 hrs followed by laser irradiation at wavelengths 594 and 682 nm, respectively. Untreated A375 (cell + 0 PS, 0 LI) were compared with those treated with 2.5 *μ*M PS, 5 J/cm^2^ and those treated with 2.5 *μ*M PS + 1, 2.5, 5, and 10 J/cm^2^.

The amount of ATP was found higher in laser-treated cells. Cells incubated with PSs at 2.5 *μ*M and those treated with 5 J/cm^2^ alone did not result in significant loss of metabolically active cells. However, exposure of A375 cells to Hyp-PDT and AlPcS_4_Cl-PDT resulted in a decreased ATP signal. The decrease in ATP content of cells was laser dose-dependent (Figures 6, 7, and 8). The A375 cells after PDT treatment displayed significant changes in cell proliferation as compared to untreated cells.

When comparing the reduction in proliferation between the control and Hyp-PDT at different fluences, it varies in efficiency in reducing cellular activity compared to AlPcS_4_Cl-PDT although the time of exposure also influences the effectiveness of the photosensitizers.

In addition, when comparing each photosensitizer at increasing fluences to their controls receiving no photosensitizer or irradiation, there was in most cases a statistically significant decrease in cellular proliferation as indicated in the figures.

Of particular interest is to note that the time of incubation of cells with respective photosensitizer directly influenced the proliferation as expressed by ATP content. The longer the incubation time prior to irradiation, namely, 1, 4, and 24 hrs, the greater the decrease in proliferation. This may be directly related to the uptake and positioning of the photosensitizer within the cell and cellular organelles.

Within all three methods used in this study, TB, LDH, and ATP, the decreased viability, membrane integrity, and proliferation of cells were inversely proportional to the concentration of Hyp, AlPcS_4_Cl, and laser dose alone or combined. The higher the concentration of PSs or laser dose the higher the loss of cell viability, membrane integrity, and proliferation of cells.

### 3.5. Mode of Cell Death

The Annexin V FITC apoptosis detection kit was used to evaluate the mode of cell death after Hyp-PDT and AlPcS_4_Cl-PDT treatment. Untreated cells were compared to PS-treated, laser-treated, and PDT-treated cells using flow cytometry.

A375 cells treated with 2.5 *μ*M Hyp; 2.5 *μ*M AlPcS_4_Cl; 5 J/cm^2^ laser at 594 nm and laser irradiation at 682 nm displayed no significant variations in percentage viability compared to the control group. Hyp-PDT treatment resulted in a significant number of apoptotic cells, 24.5% late apoptosis and 20.6% early apoptotic cell death after 4 hrs PDT treatment and 45.1% of late apoptotic cell death and 28.3% early apoptotic cell death after 24 hrs PDT treatment. AlPcS_4_Cl-PDT treatment caused 14.3% late apoptosis and 3.1% early apoptotic and 1.2% necrotic cell death mode after 4 hrs PDT treatment and 18.2% of late apoptotic, 3.4% early apoptotic, and 3.9% necrotic cell death mode after 24 hrs PDT treatment.  Table 4 indicates the differences between Hyp-PDT and AlPcS4Cl-PDT cell populations as percentages of live, necrotic, early apoptotic, and late apoptotic cells. These results are in agreement with the results presented in TB, LDH, and ATP that indicate that duration of incubation time of cells with PS is directly related to increased levels of cell damage and death.

Overall it was noted that cells incubated with photosensitizers did not inflict significant cellular damage until irradiated. Upon irradiation, PDT, both Hyp and AlPcS_4_Cl induce early apoptosis. It is notable that Hyp also cause a significant amount of late apoptotic cells while the main mode of cell death induced by AlPcS_4_Cl is early apoptosis. Additionally, the incubation period of cells with photosensitizer after irradiation of 4 and 24 hrs influences the percentage of cell death induced in an increasing time-dependent manner.

Irrespective of incubation times, mode of cell death in A375 cells from both Hyp-PDT and AlPcS_4_ClPDT was induced as early apoptosis. Although with Hyp-PDT a significant percentage of cells was observed in the late apoptotic phase as well. Apoptosis is a complex process that involves many pathways regulated by specific proteases called caspases. Activation of caspase 3 and a collapse in plasma membrane integrity are main indicators of early apoptosis whereas late apoptosis is characterized by nuclear fragmentation. Our future work will confirm these findings by in-depth genetic expression studies.

## 4. Discussion 

PDT for treatment of skin cancer has a series of inherent advantageous properties over chemotherapy and radiotherapy based on the fact that they destroy diseased tissue while leaving normal tissue unharmed. PDT is the product of a reaction of a tumor localizing PS and light [20, 21]. The efficiency of PDT, however, depends on characteristics of the PS, wavelength of light to activate the PS, and molecular oxygen [22]. The results obtained from cell viability and cytotoxicity studies of treated A375 cells indicated that 2.5 *μ*M of both Hyp and AlPcS_4_Cl caused an approximately 50% decrease in cell viability and therefore was chosen as the optimal concentration of PSs for use in phototoxicity experiments to determine the optimal PDT treatment of melanoma* in vitro*.

The continuous wave diode lasers at wavelengths 594 nm and 682 nm were chosen to irradiate A375 cells in absence or presence of Hyp and AlPcS_4_Cl, respectively. Irradiating cells only with both lasers at 1, 2, 5, and 5 J/cm^2^ was found nontoxic to cells. However, using TB and LDH assays, contradictory results were obtained in terms of treatment with 10 J/cm^2^ at both wavelengths. With LDH method, only 10 J/cm^2^ of laser irradiation at 682 nm showed a slight damage of cell membrane to A375 cells (Table 2), as compared to results in TB method that a significant decrease in cell viability was obtained at 10 J/cm^2^ (Figure 2). Hadjur et al., in 1996, demonstrated for the first time that photoactivated Hyp under aerobic conditions is toxic to human amelanotic cells than in pigmented melanoma, while aluminium disulphonated phthalocyanine has been shown to have potential for the PDT treatment of skin melanoma cancer [13, 23]. Phototoxicity measurements in this study showed that A375 cells are susceptible to photoactivated Hyp and AlPcS_4_Cl; however, to a larger degree, cells were already affected by photoactivated Hyp from as early as one hour after cells were incubated with Hyp followed by irradiation at 594 nm (Figure 3). The viability of A375 cells decreased with the increase in PS concentration at every incubation period. The incubation of cells for 24 hrs with Hyp, followed by irradiation with corresponding laser doses, was lethal to A375 cells (Figure 8).

It is well known that the efficiency of PDT depends on the internalization of PSs in cells. The nucleus, plasma membranes, mitochondria, and lysosomes have been identified as targets for PS localization [24]. Hypericin localizes predominantly in intracellular membranes such as ER and Golgi apparatus and under different treatment conditions in the mitochondrial and nuclear membrane, as well as in lysosomes [19, 25]. On the other hand, AlPcS_4_Cl has been previously been shown to localize in mitochondria and lysosomes of melanoma cells* in vitro* [16, 17]. Studies by Castano et al., 2005, showed that PSs which localize in mitochondria induce cell damage via apoptosis, whereas those that localized in lysosome would generally cause cell damage via necrosis and apoptosis [26].

Davids et al., 2008, reported that exposure of pigmented melanoma and melanocytes to 3 *μ*M Hyp activated with UV light induces a necrotic mode of cell death and an apoptotic mode of cell death in nonpigmented melanoma cells and keratinocytes. We demonstrate, in this study, that incubation of A375 cells with Hyp for 24 hrs followed by irradiation with laser at 594 induces apoptotic cell death, whereas incubation of A375 cells with AlPcS_4_Cl for 24 hrs followed by irradiation with 682 nm laser results in low percentage of apoptotic and necrotic death mode as compared to a higher degree of apoptotic cells in Hyp-PDT-treated cells. Our results are in agreement with previous report that the type of cell death activated by PDT can be influenced by modifying the treatment protocol to lead to a desired apoptosis/necrosis ratio that is advantageous for complete tumor destruction. Additionally, aspects inducing the cell death mode include incubation settings, PS concentration and localization, and light dose [27].

## 5. Conclusion

In conclusion, the results presented in this paper show that photodynamic effect of Hyp and AlPcS_4_Cl combined with laser irradiation* in vitro* occurs as early as 1 hr after incubating cells with PS, followed by laser irradiation. Irradiation of cells in the presence of Hyp and AlPcS_4_Cl, with diode laser at 594 nm and 682 nm, respectively, induced destruction of A375 cells in a PS concentration and time- and light dose-dependent manner. The longer the incubation period of cells with PS, the higher the PDT effect. A375 cells were found to be more susceptible to photoactivated Hyp as compared to AlPcS_4_Cl. Hyp is shown to be the hopeful candidate for the desirable PDT destruction of melanoma* in vitro*. PDT with Hyp or AlPcS_4_Cl has been shown to successfully induce apoptosis or necrosis in melanoma.

## Figures and Tables

**Table 1:** Parameters to determine optimal concentration of PS and optimal laser dose on A375 cells.

Parameters	Laser treatment: dose response		PS treatment: optimal concentration		
Wavelength	594 nm	682 nm	PS	[AlPcS_4_Cl]	[Hyp]
Wave emission	Continuous	Continuous	Concentration	0 *µ*M	0 *µ*M
Spot Size	3.3 cm^2^	3.3 cm^2^		1 *µ*m	1 *µ*m
Output power	124 mW	46 Mw		2.5 *µ*M	2.5 *µ*M
Power density	13.658 mW/cm^2^	5.067 mW/cm^2^		5 *µ*M	5 *µ*M
Laser fluence (exposure times)	0 J/cm^2^ (0 min, 0 sec)	0 J/cm^2^ (0 min, 0 sec)		10 *µ*M	10 *µ*M
	1 J/cm^2^ (1 min, 0 sec)	1 J/cm^2^ (3 min, 29 sec)			
	2 J/cm^2^ (3 min, 08 sec)	2 J/cm^2^ (8 min, 22 sec)			
	5 J/cm^2^ (6 min, 15 sec)	5 J/cm^2^ (16 min, 04 sec)			
	10 J/cm^2^ (12 min, 30 sec)	10 J/cm^2^ (32 min, 00 sec)			

**Table 2:** LDH membrane integrity assay to evaluate effect of laser irradiation at 682 and 594** **nm on A375 cells.

Laser irradiation	Cells + 0 J/cm^2^	Cells +1 J/cm^2^	Cells + 2.5 J/cm^2^	Cells + 5 J/cm^2^	Cells + 10 J/cm^2^
*λ*682	0.490 ± 0.046^a^	0.474 ± 0.047	0.535 ± 0.050	0.540 ± 0.061	0.617 ± 0.095^*∗*^
*λ*594	0.505 ± 0.047	0.536 ± 0.052	0.544 ± 0.046	0.547 ± 0.046	0.676 ± 0.079

*n* = 6; LDH: Lactate Dehydrogenase; ^*∗*^*P* < 0.05; ^a^±SE.

**Table 3:** LDH membrane integrity assay to evaluate PDT effect of Hyp and AlPcS_4_Cl.

	Cells + no PS, no LI	Cells + 2,5 *µ*M PS	Cells + 5 J/cm^2^	Cells + 2,5 *µ*M PS + 1 J/cm^2^	Cells + 2,5 *µ*M PS + 2.5 J/cm^2^	Cells + 2,5 *µ*M PS + 5 J/cm^2^	Cells + 2,5 *µ*M PS + 10 J/cm^2^
AlPcS_4_Cl							
1 hr	0.460 ± 0.039^a^	0.470 ± 0.026	0.386 ± 0.026	0.485 ± 0.031	0.494 ± 0.024	0.492 ± 0.029	0.507 ± 0.031
4 hrs	0.476 ± 0.016	0.489 ± 0.014	0.445 ± 0.026	0.500 ± 0.014	0.525 ± 0.016	0.545 ± 0.020^*∗∗*^	0.534 ± 0.028^*∗∗*^
24 hrs	0.407 ± 0.018	0.448 ± 0.014	0.360 ± 0.017	0.412 ± 0.024^*∗∗*^	0.515 ± 0.035^*∗∗*^	0.524 ± 0.036^*∗∗*^	0.535 ± 0.036^*∗∗*^

Hyp							
1 hrs	0.453 ± 0.017	0.467 ± 0.017	0.428 ± 0.020	0.475 ± 0.017	0.482 ± 0.016	0.495 ± 0.018	0.562 ± 0.045^*∗*^
4 hrs	0.431 ± 0.012	0.445 ± 0.011	0.403 ± 0.018	0.461 ± 0.013	0.471 ± 0.013^*∗∗*^	0.527 ± 0.033^*∗∗*^	0.539 ± 0.035^*∗∗*^
24 hrs	0.450 ± 0.006	0.480 ± 0.009^*∗*^	0.438 ± 0.009	0.496 ± 0.010^*∗∗*^	0.500 ± 0.011^*∗∗*^	0.527 ± 0.010^*∗∗*^	0.566 ± 0.030^*∗∗*^

*n* = 6; LDH: Lactate Dehydrogenase; LI: laser irradiation; PS: photosensitizer; ^*∗*^*P* < 0.05; ^*∗∗*^*P* < 0.01; ^a^±SE.

**Table 4:** The Annexin V FITC apoptosis detection kit was performed to evaluate mode of cell death on A375 cells after treatment with Hyp-PDT or AlPcS_4_Cl-PDT.

Variable	Mode of cell death			
	Live cells (%)	Necrotic cells (%)	Early apoptotic cells (%)	Late apoptotic cells (%)
Hyp				
Cells only	100	0	0	0
Cells + 5 J/cm^2^ at 594 nm	92.7	2	2.7	2.6
Cell + Hyp (2.5 *µ*M)	93.7	0.4	4.2	1.7
Cells + PDT 4 hrs	53.5	1.4	24.5	20.6
Cells + PDT 24 hrs	25	1.6	45.1	28.3

AlPcS_4_Cl				
Cells only	100	0	0	0
Cells + 5 J/cm^2^ at 682 nm	92.7	1.5	3.1	2.7
Cells + AlPcS_4_Cl (2.5 *µ*M)	87.5	0.4	9.9	2.2
Cells + PDT 4 hrs	81.3	1.2	14.3	3.1
Cells + PDT 24 hrs	74.5	3.9	18.2	3.4
